# Formulation and Clients’ Agency in Cognitive Behavioral Therapy

**DOI:** 10.3389/fpsyg.2022.810437

**Published:** 2022-03-10

**Authors:** Xueli Yao, Boyu Dong, Weining Ji

**Affiliations:** School of Foreign Languages, Qingdao Agricultural University, Qingdao, China

**Keywords:** formulation, agency, cognitive behavioral therapy, conversation analysis, talk-in-interaction

## Abstract

The experience of loss of agency is one of the reasons for clients to go for psychotherapy. Enhancing clients’ agency has been considered a fundamental factor for successful treatment in psychiatry and psychotherapy, yet few studies have investigated the interactional realization of how therapists do this in authentic psychotherapeutic encounters. Drawing on audio-recorded talk-in-interaction between clients and psychotherapists in cognitive behavioral therapy (CBT) encounters at a mental health center in China, this paper uses the method of conversation analysis to demonstrate how therapists ascribe agency positions to clients by issuing formulations of what the clients have just said. Two types of formulation were identified: affirmative formulations and challenging formulations. In the first type, the therapists highlight the positive aspect of the clients’ description of their experiences and ascribe an agentic position to the clients. In the second, the therapists challenge the clients’ implausible views and their non-agentic positioning of themselves. This study shows that the therapists’ formulation could be employed to manage the epistemic difficulties associated with claiming knowledge about the clients’ inner states and assessing their feelings. In this sense, the formulation is a robust interactional device in negotiating epistemic problems in addressing the clients’ experiences and promoting their agency in therapy. However, it is noteworthy that in the challenging formulation, therapists claim privileged access to the clients’ knowledge domain and challenge their prior epistemic status, which might run the risk of engendering clients’ resistance.

## Introduction

Agency is one of the central issues in psychotherapy. Generally, it refers to the clients’ ability to attribute thoughts, feelings, and actions to themselves as well as their capability to take initiative and responsibility for their own actions in everyday life (Avdi, 2005; Etelämäki et al., 2021). Previous research reports that the experience of loss of agency is one of the reasons for clients to go for psychotherapy, thus enhancing the clients’ agency has been recognized as critical in facilitating therapeutic change (Wahlström, 1990, 2006; Williams and Levitt, 2007; Etelämäki et al., 2021).

Therapists and researchers alike stress the importance of agency in psychiatry and psychotherapy. Promoting the clients’ agency is considered a fundamental factor for successful treatment across different psychotherapeutic approaches (Hoener et al., 2012). For instance, McWilliams (1999, p. 15) reports that helping clients be aware of their agentic capacities “takes precedence over most other considerations” in psychodynamic psychotherapy. Bohart (2000) states that, in humanistic therapies, mobilizing the clients’ agency is an effective way to achieve improvement and heal themselves.

Accordingly, various methods and principles that help develop clients’ diminishing agency are explored across different psychotherapeutic traditions. For instance, in humanistic therapies, introspective self-examination is understood as a method to develop the clients’ personal perspective and responsibility (Rogers, 1951; Williams and Levitt, 2007); in psychoanalysis, there is a belief that dealing with client resistance plays an important role in enhancing the clients’ agency (Beutler et al., 2002) while in cognitive-behavioral therapies, altering distorted thought patterns and gaining new skills are seen as efficient ways to activate and promote clients’ agency (Hollon and Beck, 1979; Williams and Levitt, 2007).

Although the previous studies are firm in their assertion that agency is significant and despite the efforts they have made in promoting the clients’ agency, they are less helpful in saying how it is to be done in that few of them have investigated the interactional realization of how therapists do this in authentic psychotherapeutic encounters. As compensation for this deficiency, recently, there is a small but growing amount of literature concerning the study of client-agency by the method of conversation analysis (CA) (Schegloff, 1968, 2007), which carries out fine-grained observational analysis of naturally occurring psychotherapeutic talk-in-interaction.

Conversational analytic studies on client agency suggest that agency is a social activity that can be constructed and negotiated through talk-in-interaction (Avdi, 2005; Enfield and Kockelman, 2017; Etelämäki et al., 2021). In primary care, Koenig (2011) and Hultberg and Rudebeck (2017) explore agency through patient resistance to doctor’s treatment recommendations and they argue that patients’ non-acceptance may be used as an important interactional resource to promote patient agency in treatment decision-making. In this sense, agency is rarely considered as a possession of an individual, but as a series of meaningful actions that emerge through the interaction (Boden, 1990; Koenig, 2011). This view of agency corresponds with the methodology of CA, which analyzes in detail the concrete practices and actions that participants employ to achieve the outcomes at the turn-by-turn level.

Similarly, in psychotherapy, researchers who adopt this view of agency tend to analyze the dynamic construction of agency through the employment of linguistic practices, such as personal references (Kurri and Wahlström, 2007; Etelämäki et al., 2021). In their study, Kurri and Wahlström (2007) examine the development of a therapist’s formulation of her client’s agentless problem narration. They show that the client may use agentless talk, i.e., impersonal constructions, as a strategy to escape responsibility while the therapist employs it to save the client’s moral face. In the study of Etelämäki et al. (2021), they advance the analysis of agency by extending it to the clients’ emotions and experiences. Through the examination of personal forms used by therapists, they display that zero-person does not necessarily connect with a weak agency; instead, it may be used for strengthening the clients’ agency in the sense of control and responsibility in the long run. Thus, this framework lays emphasis on the role of linguistic practices in the process of displaying and constructing the clients’ agency positioning (Kurri and Wahlström, 2007; Toivonen, 2019; Etelämäki et al., 2021).

Building upon the previous conversation analytic research on agency, the study at hand views agency as a social activity that can be negotiated through real-time talk-in-interaction between the therapist and the client. Specially, we characterize the clients’ agency as having several components, including the ability to make self-reflection and self-regulation, and the competency to take initiative and responsibility for their actions in the everyday life. In this study, we analyze the negotiation and construction of clients’ agency positions in psychotherapy through the use of the conversational practice, namely, *formulation*. By focusing on the therapists’ formulation of the clients’ problem statements, we discuss the ways in which the therapists ascribe agentic or non-agentic positions to the clients.

In what follows, we begin by shortly introducing the term *formulation* and the previous research on how this sort of practice is used in the psychotherapeutic context. This will form the basis for our analysis of the study, presented in the section “Results”.

## The Formulation in Previous Research

Formulation, first proposed by Garfinkel and Sacks (1970, p. 350) in their classic work *On Formal Structures of Practical Actions*, refers to a conversational practice whereby participants in interaction may “describe that conversation, or explain it, or characterize it, or explicate, or translate, or summarize, or furnish the gist of it, or take note of its accordance with rules, or remark on its departure from rules.” Later, researchers dropped away Garfinkel and Sacks’ observation (1970) of such a phenomenon as *where-we-both-are-in-the-conversation* and are in favor of Heritage and Watson (1979) narrower and more specific version: a practice of proposing a version of events which follows directly from the other person’s own account, but introduces a transformation to some degree. Most research in CA has adopted this narrower use of formulation (e.g., Drew, 2003; Antaki et al., 2005; Hutchby, 2005; Antaki, 2008; Weiste and Peräkylä, 2013).

Heritage and Watson (1979) further identified two types of formulation: gist formulation and upshot formulation. Gist formulation constitutes clarification or demonstrations of comprehension with the talk thus far while upshot formulation presupposes some unexplicated version of the gist or extracts an implication from what has been said. Through formulating, one participant puts the previous talk into words, thereby pinpointing the upshot or gist of it, and at the same time, transforming it by selecting certain parts and deleting others (Heritage and Watson, 1979; Drew, 2003; Antaki, 2008).

Therapeutic formulations have been a topic of great concern in conversation analytic study since Davis (1986) first explored a therapist’s formulation of the client’s talk. She demonstrated how the formulation is deployed by the therapist as a way to transform the client’s “non-psychological” troubles into a typical therapy problem calling for professional psychotherapeutic intervention. Ever since Davis’ work, an array of CA studies on psychotherapy show that formulations may be used for multiple interactional purposes. For instance, they can transform the raw material of the clients’ talk into psychological issues suitable for therapeutic work. Formulations can also be used to shape the clients’ symptoms and manage the progress of therapeutic sessions (Hak and de Boer, 1996; Antaki et al., 2005; Hutchby, 2005; Muntigl, 2007; Antaki, 2008; Weiste and Peräkylä, 2013).

Previous CA researchers have pointed out that although formulation is about summarizing, explaining, or giving the gist or upshot of what has been said by the client, it is not in fact entirely neutral and is rarely undertaken for its own sake; rather, it can be tendentious in that it focuses on some particular element of the previous talk and preserves that element as the topic for further talk (Heritage, 1985; Hutchby, 2005; Weiste, 2016). This characteristic makes formulation an important interactional resource for psychotherapists to transform clients’ accounts into a problem that can be therapeutically addressed (Hutchby, 2005; Weiste, 2016), or to shepherd the clients’ presentation toward subsequent therapeutical interpretation (Antaki et al., 2005; Antaki, 2008). Such transformation and direction could entail negotiation about the meaning and significance of the client’s experience. The result of the negotiation is significant for exploring the thoughts, feelings, and behaviors of clients, which could contribute to increasing their well-being and achieving higher levels of functioning (Versteeg and te Molder, 2016; Peoples et al., 2020).

In the present study, we focus on the types of formulation employed by therapists to address the meaning and significance of what the clients have said. Specifically, we deal with how the therapists ascribe agentic or non-agentic positions to their clients by issuing formulations of the clients’ feelings-talk in the problem statement. Although any therapy or therapist may attach importance to the promotion of clients’ agency, in this study, we focus on the management of clients’ agency in cognitive behavioral therapy (CBT). In CBT, the clients’ problem statement is underscored as a crucial site to achieve a shared understanding of the clients’ problems and perspectives (Beckwith and Crichton, 2010) and it highlights the importance of the clients’ ability to influence their own health through changing misperceptions and gaining new skills. Thus, the study at hand explores the cognitive-behavioral therapists’ use of the formulation as a response to the clients’ problem statements. More specifically, by focusing on the turns of the therapists’ responsiveness to the clients’ problem talk, the study examines how the therapists ascribe agency positions to their clients through reshaping the clients’ descriptions of their problems and how the CBT therapists orients to the clients’ agency in their following turns.

## Data and Methods

The data for the article is part of an ongoing larger study investigating the interactional practices in CBT in China. All data were collected by way of fieldwork in a mental health center in the north of China from 2017 to 2019 under the approval of both the therapists and the clients. In the larger database, routine sessions involving 13 clients at the center were audiotaped over their full courses of treatment with four therapists. We randomly selected the first three sessions of CBT in the therapy of four clients as data for this article. The first three sessions of each client were chosen for analysis because the clients’ experiences and agentic positions were typically assessed and negotiated through these sessions.

The selected 12 audio-recorded sessions, involving approximately 11 h, were from four different dyads run by two professionally qualified cognitive-behavioral therapists (two males). Four clients participated: two female clients, one suffered from depression and the other from obsessive compulsory disorder; two male clients, one suffered from schizophrenia and the other from anxiety neurosis. The therapists and their clients met once a week and each session lasted from 45 to 55 min.

The analysis employed audio recordings and transcripts as the primary data, and the first author in this study was also the data collector of this sub-corpus. The audio-taped interactions were analyzed by using CA which takes the naturally occurring episodes of talk-in-interaction as data (Schegloff, 1968). The data were transcribed using the transcription system developed by Jefferson (2004) (Transcription notations see Appendix). Anonymity was used to secure the privacy of all the participants.

The transcript has three lines which include the Chinese *Pinyin*, a word-by-word gloss, and an idiomatic translation. The first line of data is presented in Chinese *Pinyin*. In the second line, the utterance is glossed English word by word which will help the reader to know what is happening in the Chinese original. In the third line, there is the idiomatic translation of the original Chinese. Considering the potential for interlinguistic and intercultural issues that may be relevant to the translation of the transcript and the clinical setting, we acknowledged that it is not easy to translate the transcript from one language to another. To minimize these problems, we asked help from two scholars in this field who can speak both Chinese and English to polish the translation. Phonetic and prosodic features such as length of silence, pauses, stress, intonation, and vocalic lengthening were transcribed. Besides, the punctuation symbols are not used grammatically, but to indicate the intonation contours of talk.

In order to explore how the therapists ascribe agentic or non-agentic positions to clients in their responses to the clients’ problem talk, two steps were employed in the study at hand. In the first step, the instances where the therapists formulated the clients’ problem talk were identified from the transcripts. The formulation is understood according to Heritage and Watson (1979) definition: an utterance that displays an understanding of the previous speaker’s talk by introducing an altered version of it. The search identified 49 such instances, among which 27 instances contain the exploration of the clients’ agency positions. In the second step, the 27 instances were analyzed in the local context of interaction. Especially, three aspects were examined: which include the clients’ problem statement, the therapists’ formulation, and the turns and agendas following the formulation.

## Results

In the responses to the clients’ problem talk, we found that therapists regularly made use of two types of formulation to ascribe agentic or non-agentic positions to their clients: affirmative formulation and challenging formulation. Among them, affirmative formulation (MacMartin, 2008) usually occurred in the sequential environment where the therapists formulated the positive aspect in clients’ problem talk (this occurred in 11 out of 27 instances); while challenging formulations were typically used in the sequential environment where therapists showed disagreement with clients’ presentation of their problems or experiences, particularly with the statement of their implausible beliefs or misbehaviors (in 16 cases). The four data extracts discussed in the following are the most illustrative and representative of the variation within the categories.

### Affirmative Formulation: Endorsing Clients’ Competence and Agency

Affirmative formulation emphasizes the positive aspect of the clients’ problem account and highlights the clients’ agency to deal with these problems or to achieve improvements (Stommel and van der Houwen, 2013). Extract 1 contains an example. In the extract, the client, a 45-year-old woman with depressive symptoms, is reporting to the therapist how she managed the depressive situation when she was at home the other day. In all data extracts, therapists are referred to as TH and clients as CL. The turns that contain the therapists’ formulation are indicated by arrows.

**Extract 1**


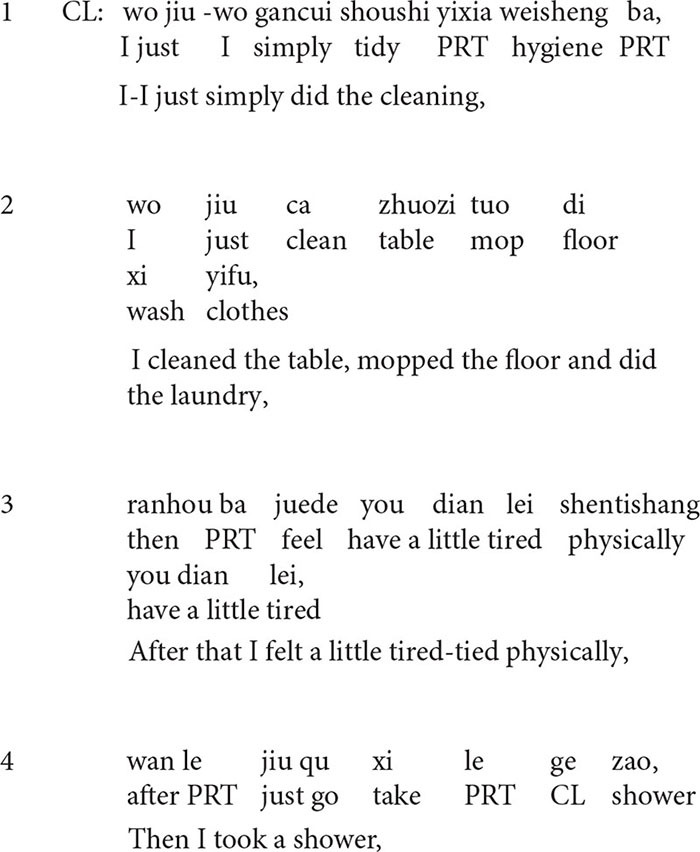



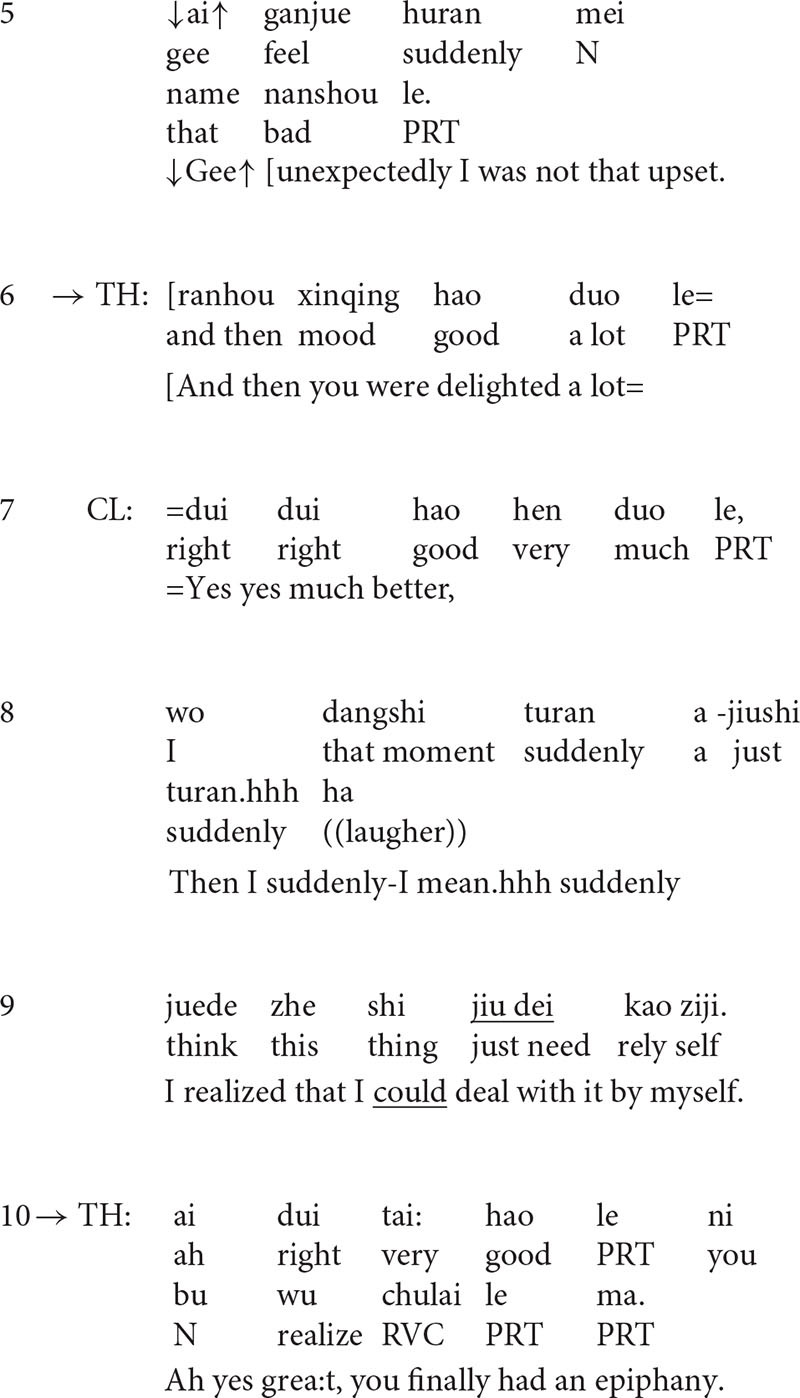


In this extract, from lines 1–4, the client describes what she did to manage the depressive mood: she did some cleaning, including cleaning the table, mopping the floor, doing laundry, and taking a shower. In line 6, the therapist produces an upshot formulation of the client’s description: *and then you were delighted a lot*, which overlaps with the client’s turn in line 5. The turn-initial particle *and then* (“ranhou”) in line 6 shows that the formulation is a valid understanding inferred from the preceding talk (Bolden, 2010), but it transforms the frame of the talk by deleting some issues: *what the client did*, and highlighting the others: *the positive implication of what she did.* The formulation is confirmed by the client immediately. She produces two *yes* (“dui dui”) in a row and then upgrades her own evaluation of the experience from *not that upset* (line 5) to *much better* (line 7).

In line 8, the client continues describing the improvements she made in the management of the situation: s*he realized that she could deal with it (i.e., get rid of her depression) by herself.* In the following turn, the therapist makes a positive assessment by producing a prolonged *great*, and then he endorses the client’s agency by upgrading *realized* (line 9) into *had an epiphany* (line 10). One of the psychoeducational goals in the sessions has been to make the client realize that she should depend on herself to get rid of anxiety and depression. For the client, the process of making therapeutic changes is like *having an epiphany* (“wu”). *Wu*, which refers to realizing something like having an epiphany, demonstrates the process that the client comes to self-consciousness and self-awareness. In this formulation, the client is formulated as an active agent in her achievement of changes.

Extract 2 is another example that contains affirmative formulation. The client is a 37-year-old man suffering from schizophrenia and can hear voices (probably hallucinations). As a response to the therapist’s inquiry (data not shown), the client provides a presentation of his current situation: he still hears the voices occasionally. Core symptoms of schizophrenia are hallucinations that may have a significant and complex bearing on the agency.

Unlike the client from Extract 1, the client in Extract 2 does not present his experience with a completely positive stance. However, despite this, the therapist finds some positive side in the client’s presentation and endorses his agency (although limited) to deal with his problem.

**Extract 2**


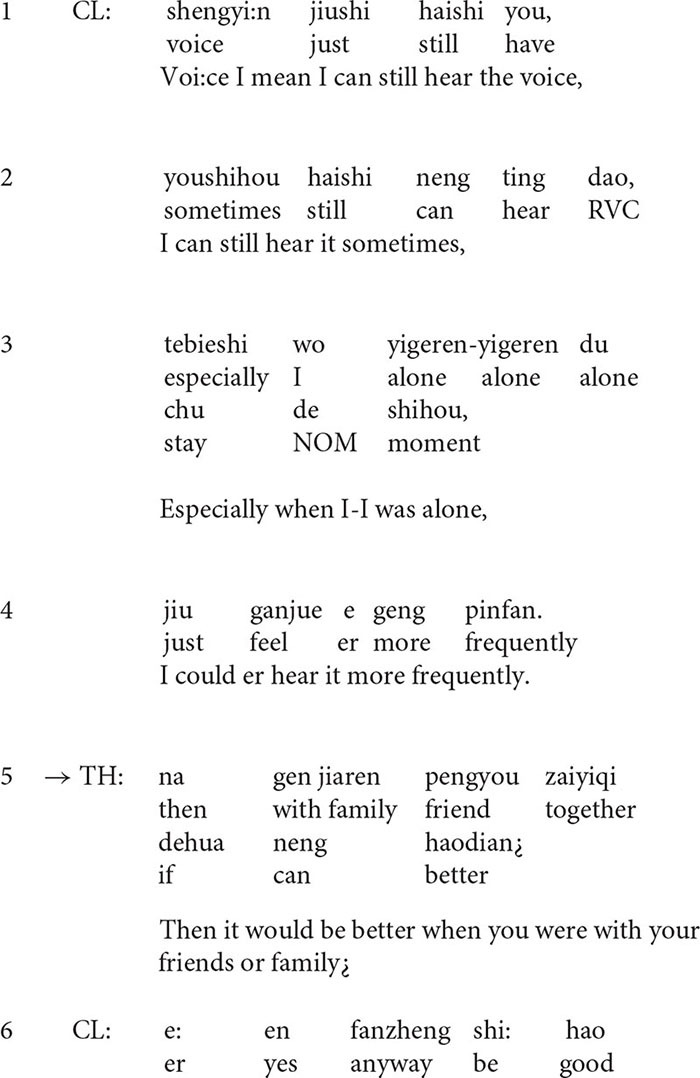



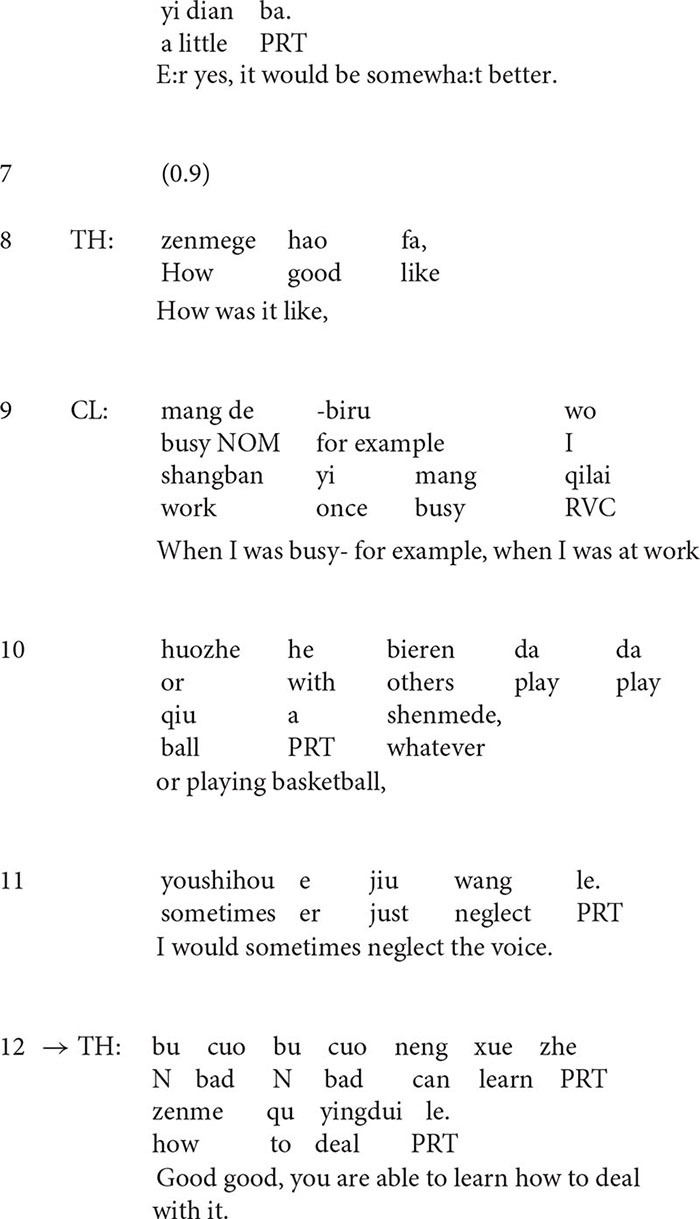


At the beginning of the extract (lines 1–4), the client describes his situation with a rather negative stance (Stivers, 2008). He occasionally hears voices and the situation gets worse when he stays alone. However, in line 5, the therapist’s formulation transforms the negative perspective by finding something positive in the presentation. The turn-initial particle *Na* (“then” in English) suggests that the turn is inferred from the client’s previous talk, but the therapist makes some transformation, i.e., he transforms *when the client was alone, the situation got worse* into *when he was with his friends or family, the situation would be better*, thus highlighting the positive side of the story. In line 6, the client confirms the formulation although he tones down the positive implication *better* by downgrading it into *somewhat better.*

The affirmative formulation deflects the course of talk to the positive side and it seems to encourage the client to say more (Tiitinen and Ruusuvuori, 2014) about the positive aspect of the situation. The follow-up question in line 8 provides evidence for this. In line 9, the client lists examples of such occasions, for instance, when he was *busy at work* or *playing basketball*, he may *neglect the voice*. As a response to the statement, in line 12, the therapist produces another formulation in the extract which highlights the clients’ ability and agency to cope with the situation.

In sum, we found that when the clients described their problems or experiences with a positive stance, the therapists highlighted the positive perspective; when the clients’ perspective is not explicitly positive, the therapists would nonetheless find the potential or hidden positive side in clients’ problem talk, underscoring their skills or abilities (although it may be very limited skills). After confirmation from the clients, the therapists generally provided positive feedback or evaluation of the clients’ agency position or abilities to manage the situation.

### Challenging Formulation: Tackling the Clients’ Dysfunctional Patterns

Clients with severe mental problems such as schizophrenia, anxiety disorder, and obsessive-compulsory disorder tend to have some unreasonable beliefs or misbehaviors which may lead to fear and anxiety. CBT practitioners emphasize the importance of uncovering clients’ implausible views to enhance their awareness, reflection, and agency (Murray et al., 2008). However, few studies investigated how it is realized in authentic therapeutic encounters.

In this study, we found out that challenging formulations were typically used in the sequential environment where therapists showed disagreement to the clients’ presentation of their problems or experiences, particularly to the statement of their implausible views or misbehaviors. In psychotherapy, challenging does not necessarily imply aggression or hostility. It can be an effort to make clients aware of behaviors or thoughts they had not recognized (Orlinsky et al., 2004; Wright et al., 2006). This type of formulation is constructed to challenge the clients’ prior problem talk by rephrasing it as something that is obviously unreasonable.

Extract 3 contains such an example. In this extract, the client, a 26-year-old young man who suffers from anxiety neurosis, reports to the therapist that he is always in a bad mood and feels depressed because he thinks that, compared with his peers, he is a total loser (lines 1–5).

**Extract 3**


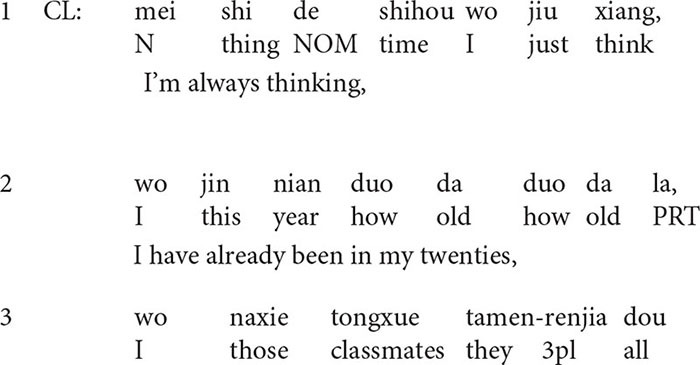



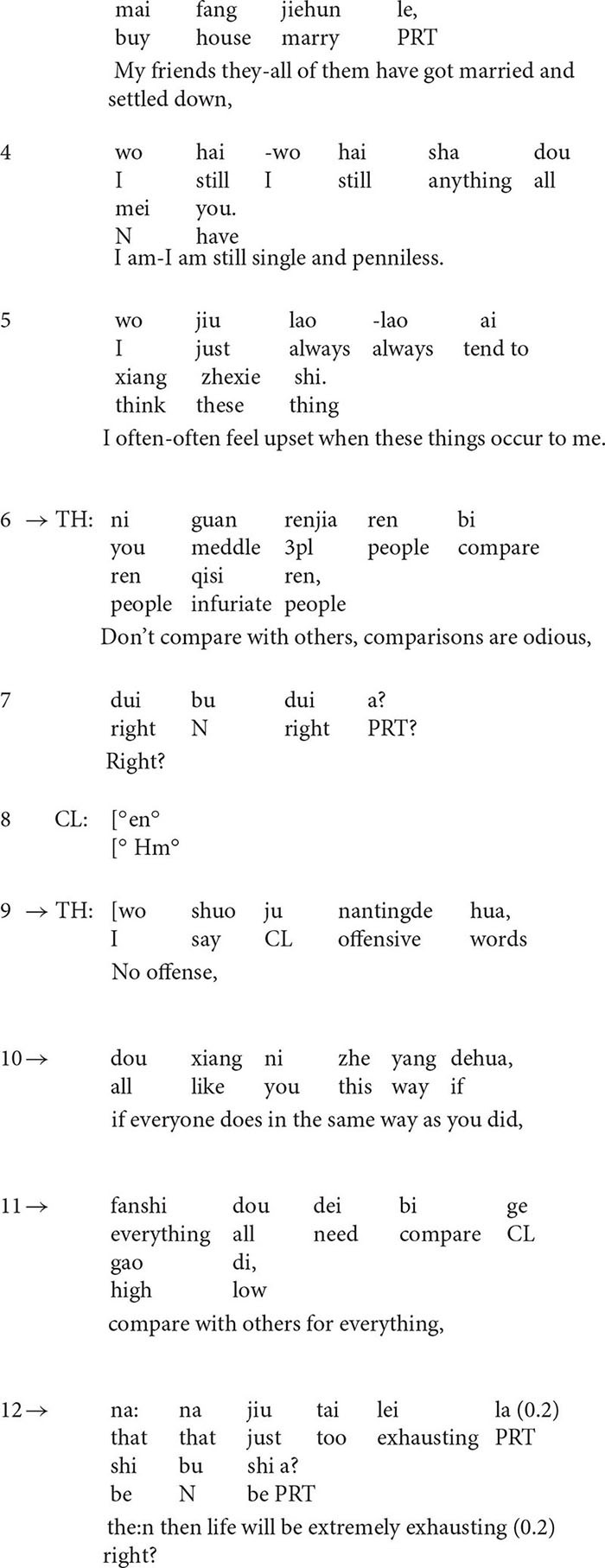



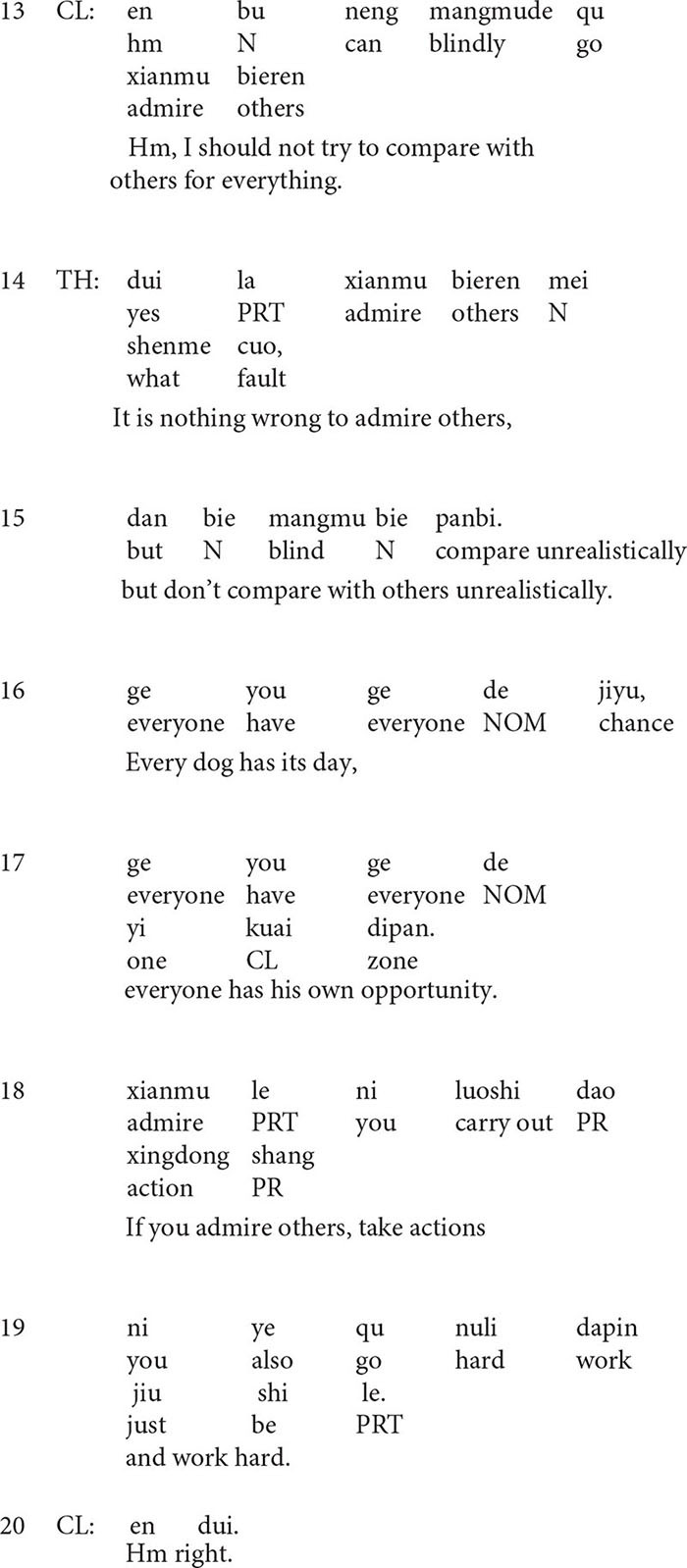


In the above extract, the person reference employed by the client in line 3 is interesting. At the turn beginning, he uses a third-person reference *my friends*, which is an unmarked non-recognitional locally initial reference form in locally initial position (Stivers, 2007). However, he immediately replaces the unmarked form, my friends, with *they* and *all of them*, both of which are the marked locally subsequent form in the locally initial position. The marked usage of the person reference form does more than just reference. In this case, the client uses *they* and *all of them* to distance him from his friends, thus building a sharp contrast between them. In addition, the extreme case formulation (Pomerantz, 1986) *all* in line 3 helps in emphasizing the sharp contrast.

In line 6, the therapist shows disagreement with the client’s behavior: *Don’t compare with others*, and then he makes an evaluation: *Comparisons are odious*. In lines 9–12, he makes an upshot formulation of the client’s ideas and behavior: *Compare with others for everything.* It is interesting that the challenging formulation is preceded by *no offense*, which in fact insinuates that what is coming will probably be offensive. Syntactically, the formulation is designed as an if-then clause: If *everyone compares with others for everything* then *life will be extremely exhausting*. The employment of the extreme case formulations *everyone*, *everything*, and *extremely* imply the implausibility and ridiculousness of the client’s perceptions, thus challenging the client’s dysfunctional patterns and projecting a disagreeing response from him. The client’s response in line 13, *I should not try to compare with others for everything* displays his uptake of the therapist’s challenging formulation. Then the psychiatrist approves the client’s response (line 14) and takes it as an opportunity to do psychoeducation and give suggestions for the client’s future actions (lines 15–19). In these suggestions, through the use of the second person reference *you* (Kurri and Wahlström, 2007; Etelämäki et al., 2021) and active verb phrases as *take action*, *work hard*, the therapist depicts the client as an active actor who has the (potential) ability to make changes.

Extract 4 provides another example. In this extract, the client, a 27-year-old woman who is suffering from obsessive-compulsory disorder for more than 2 years, is talking with the therapist about her extreme fear of germs. Before the extract (data not shown), she told the therapist that her situation was worsened after she visited a relative of hers who had a diagnosis of cancer. She is haunted by the idea that she would be infected by her relative’s “cancer germs.”

**Extract 4**


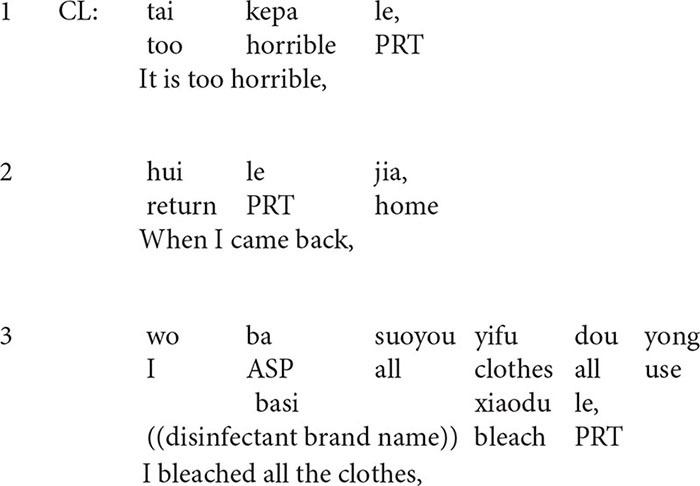



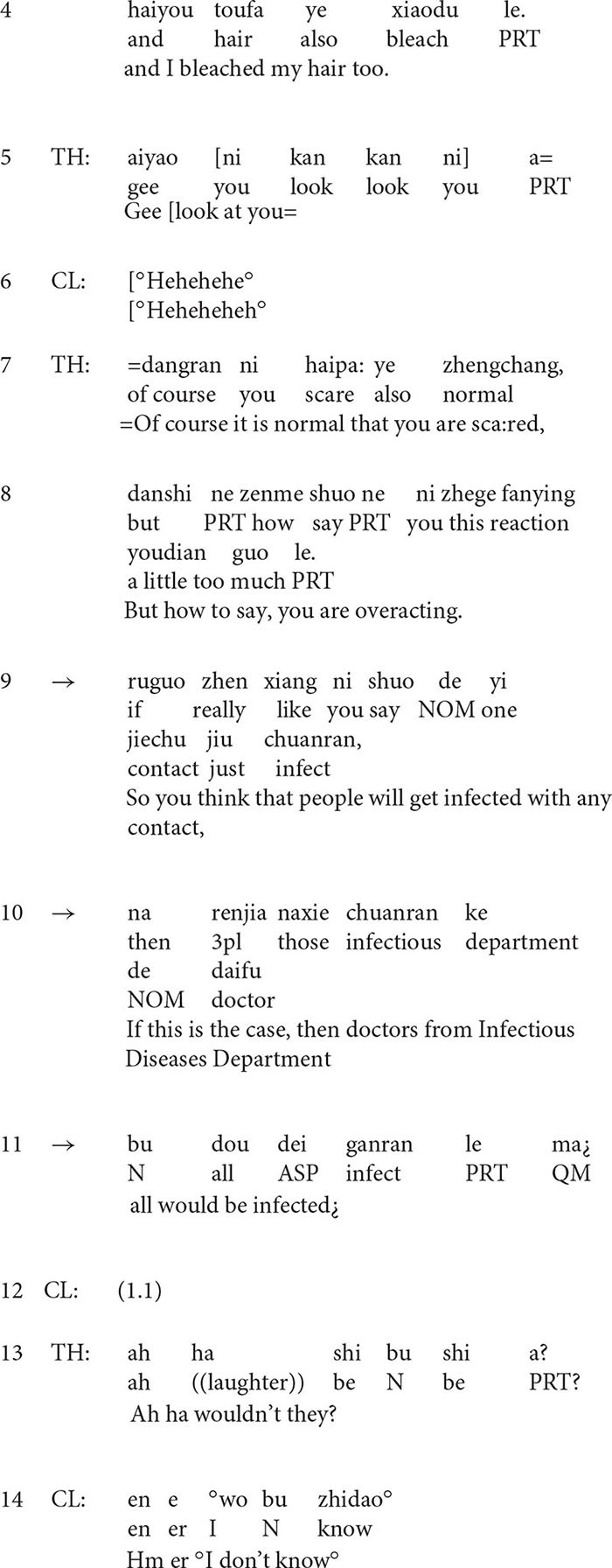


From line 7 to line 11, as a response to the client’s statement, the therapist normalizes the client’s emotional experience before claiming that the client is overacting. Then he organizes his formulation of the client’s implausible ideas in an if-then clause: *if people get infected with any contact, then all the doctors from Infectious Diseases Department would be infected.* In this formulation, the therapist, first of all, supposes that the client’s way of thinking is right, and then he makes an unreasonable inference under this condition: *all the doctors from the Infectious Diseases Department would be infected*. By making the unreasonable inference and the employment of extreme case formulations *any* and *all* (Pomerantz, 1986), this formulation is designed to project disagreement from the client.

However, in line 12, the client says nothing but keeps silent. In addition, the laughter in line 13 is worth noticing. Previous studies reveal that in medical settings, laughter is employed as an interactional resource and it is used for purposes other than amusement (Zayts and Schnurr, 2011). Similarly, the laughter in line 13 is not employed for amusement but probably for indicating the absurdity of the client’s beliefs and behaviors. After the laughter, the post-positioned queries *right* in a rising intonation invite the client’s response. Nonetheless, the client only produces a knowledge disclaimer (Weiste, 2015) *I don’t know* in a low and soft voice, and withdraws from further discussion, which may be regarded as a passive resistance of the client (Stivers, 2005; Koenig, 2011; Yao and Ma, 2017).

The examples in the above two extracts suggest that when challenging clients’ unreasonable ideas and misbehaviors, therapists are likely to, first of all, suppose that the clients’ way of thinking is right, and then they make an unreasonable inference under that condition. Therefore, linguistic patterns such as *if-then clauses* and extreme case formulations are usually employed to help in challenging the implausible ideas of the clients. Sequentially, in challenging formulation, therapists redesign the clients’ problem descriptions and statements in such a way as to elicit disagreement from them. Thus, it can be said that challenging formulation does not necessarily project confirmation, it may project disagreement from clients. However, we acknowledge that it might run the risk of engendering clients’ resistance (as in Extract 4) when the challenging formulation is employed.

In sum, when clients described their unreasonable perceptions or dysfunctional patterns, the therapists would challenge the talk by rephrasing it as something that is obviously implausible or even ridiculous. After the formulation, i.e., in the post-formulation turn, therapists usually gave suggestions for the client, aiming to help them deal with the problems. In the formulation of these suggestions, the clients were generally portrayed as an active and able agent who had or would have the ability to manage the situation.

## Discussion and Conclusion

In this article, we have described how CBT therapists ascribed agency positions to their clients by issuing formulations of what the clients have just said in their problem talk. In the interactional practice of formulating, the therapists addressed the clients’ preceding problem-indicative turn by focusing on the emotion-relevant aspects of it in the formulation.

Especially, two types of formulation were identified: affirmative formulations and challenging formulations. The affirmative formulation was employed when the clients took a positive stance toward their experiences. On such occasions, the therapists formulated the positive side of the description and usually provided positive feedback on how well the clients managed the situation under discussion. In this case, the clients were formulated as active agents and thus were ascribed to an agentic position (as in Extract 1 and Extract 2). Different from affirmative formulation, the challenging formulation was used by the therapists when the clients took a negative emotional stance toward their own experiences. In challenging formulation, the therapists challenged clients’ previous talk by transforming it into something that is apparently implausible, thus challenging the clients’ dysfunctional thoughts and their non-agentic position (as in Extract 3 and Extract 4).

Previous research has shown that therapists’ formulations are central practices used for managing clients’ problem talk (e.g., Antaki, 2008; Stommel and van der Houwen, 2013; Thompson, 2013). For instance, Thompson (2013) found that psychiatrists displayed understanding through formulating the implicit emotional and psychological meanings of clients’ talk, resulting in client adherence and an improved therapeutic relationship. The present article contributes to the previous research on formulations by exploring the ways therapists used to address the meaning and significance of clients’ feelings and experiences and ascribe agency positions to them through formulation. This is a complicated interactional agenda because typically, speakers claim epistemic priority about their own feelings and experiences (Heritage and Raymond, 2005). By employing formulation, the therapists managed the epistemic difficulties associated with claiming knowledge about the clients’ inner state and assessing their feelings. In this sense, the formulation is a robust interactional device for negotiating epistemic problems in addressing clients’ feelings and experiences.

However, it is noteworthy that formulations of clients’ agency position were designed with varying degrees of empathy (Muntigl and Horvath, 2014): affirmative formulations respected clients’ epistemic primacy (Heritage, 2013) and ceded epistemic authority to the clients, whereas challenging formulations claimed privileged access to clients’ knowledge domain and challenged the clients’ prior epistemic status (Heritage, 2013), which might run the risk of engendering clients’ resistance. More studies are needed to explore when therapists should push and when to retreat on such occasions. It is also very likely that other forms of therapy may use formulation to manage clients’ agency, but it is left for future research to document how these are realized differently or similarly. In addition, the use of audio recordings for exploring face-to-face interactions is limiting because clients’ non-verbal conducts and embodied activities are unavailable to the analysts.

To conclude, this study demonstrated how CBT therapists ascribe agency positions to their clients by issuing formulations of the clients’ problem talk through fine-grained conversational analysis of naturally occurring therapist-client talk-in-interaction as it folds. The strength of the conversation analytic methodology adopted in this study lies in their efforts to explore agency as a social activity that can be understood and negotiated in the context of the previous speakers’ turn (Peräkylä, 2013; Weiste, 2015). The findings of the research may complement therapists’ professional stock of interactional knowledge (Peräkylä and Vehviläinen, 2003) with which therapeutic work gets accomplished.

## Data Availability Statement

The raw data supporting the conclusions of this article will be made available by the authors, without undue reservation.

## Ethics Statement

Ethical review and approval was not required for the study on human participants in accordance with the Local Legislation and Institutional Requirements. The patients/participants provided their written informed consent to participate in this study.

## Author Contributions

XY, BD, and WJ: conception and design of the study, acquisition, analysis, interpretation of data, and drafting and revising the manuscript. All authors contributed to the article and approved the submitted version.

## Conflict of Interest

The authors declare that the research was conducted in the absence of any commercial or financial relationships that could be construed as a potential conflict of interest.

## Publisher’s Note

All claims expressed in this article are solely those of the authors and do not necessarily represent those of their affiliated organizations, or those of the publisher, the editors and the reviewers. Any product that may be evaluated in this article, or claim that may be made by its manufacturer, is not guaranteed or endorsed by the publisher.

## Appendix

### Transcription notations

(0.2) A number inside brackets denotes a timed pause.
[] Square brackets denote a point where overlapping speech occurs (beginning [and end]).
_ When a word or part of a word is underlined, it denotes a raise in volume or emphasis.
= The equals sign represents latched speech, a continuation of talk.
: Colons represent elongated speech, a stretched sound.
°° When there are two degree signs, the talk between them is markedly softer than the talk around it.
. hhh Hearable aspiration is shown where it occurs in the talk by the letter “h” – the more “hs,” the more aspiration.
. A full stop marks a falling intonation.
? A question mark marks a rising intonation.
, A comma marks a slightly rising intonation but is also used to indicate “continuing” intonation.
> An upside-down question mark is used for intonation which rises more than a slight rise (,) but is not as sharp a rise as for a question mark.
- A hyphen after a word or part of a word indicates a cut-off or self-interruption.
↑↓ They denote marked upstep/downstep in intonation.

## Abbreviations

ASP: aspectual marker
be: BE verbs (shi)
CL: classifier
N: negation
NOM: nominalizer (de)
QM: question marker
PR: preposition
PRT: particle
RVC: resultative verb complement
3pl: third person plural pronoun.
